# Neural and Behavioral Predictors of Speech Motor Sequence Learning in Primary Progressive Aphasia

**DOI:** 10.1162/NOL.a.281

**Published:** 2026-09-04

**Authors:** Hilary E. Miller, Michael Brickhouse, Daisy Hochberg, Alfonso Nieto-Castanon, Jason Tourville, Bradford C. Dickerson, Frank H. Guenther

**Affiliations:** Department of Speech, Language & Hearing Sciences, Boston University, Boston, MA, USA; Department of Speech, Language, and Hearing Sciences, University of Connecticut, Storrs, CT, USA; Frontotemporal Disorders Unit, Department of Neurology, Massachusetts General Hospital & Harvard Medical School, Charlestown, MA, USA; Martinos Center for Biomedical Imaging, Department of Radiology, Massachusetts General Hospital, Charlestown, MA, USA; Department of Biomedical Engineering, Boston University, Boston, MA, USA

**Keywords:** cortical thickness, Gradient Order Directions Into Velocities of Articulators (GODIVA) model, primary progressive aphasia (PPA), speech motor sequence learning

## Abstract

This study investigated the clinical and neural correlates of speech motor sequence learning in primary progressive aphasia (PPA) to test specific predictions regarding the roles of the left posterior inferior frontal sulcus (pIFS) and left ventral premotor cortex (vPMC). A cohort of 26 participants with PPA completed a 2-day non-native consonant cluster learning paradigm. Participants also completed a battery of clinical speech-language assessments, including tests of phonological and motor programming ability, and T1-weighted structural MRI scans were obtained for a subcohort (*n* = 19) of participants. Mean cortical thickness measures were extracted using FreeSurfer software for each cortical region of interest. Separate statistical analyses were conducted to test for correlations between learning outcomes on the consonant cluster paradigm (both error rate and duration) with (1) clinical measures and (2) cortical thickness. Both analyses indicated a dissociation between correlates of error rate and duration: Duration was more strongly associated with sensorimotor cortex (including the left vPMC) and clinical motor speech measures, while error rate was primarily associated with phonological working memory correlates (including the left pIFS) and clinical repetition measures.

## INTRODUCTION

Speech motor sequence learning, the process by which speakers learn to produce unfamiliar syllables in a coordinated and automatic fashion, is fundamental for fluent speech production. Seminal studies of non-speech motor sequence learning have revealed a consolidation process through which smaller units of movement representing each individual element in the sequence are reorganized, or “chunked,” into a single, optimized representation (i.e., motor program) of frequently occurring sequences or subsequences (Klapp, 1995; Wright et al., 2004).

Prior studies of speech motor sequence learning have utilized a non-native cluster learning task in which English-speaking participants practice pseudowords (e.g., “*vzat*”) containing consonant clusters that do not occur in English but are permissible in another language (Cheng & Buchwald, 2021; Davidson, 2006; Masapollo et al., 2021; Segawa et al., 2015, 2019). These studies characterize participants’ learning via improvements in accuracy and/or speed—measured as the duration of the trained consonant cluster or pseudoword. Prior behavioral work using this paradigm shows a dissociation between improvements in speed and accuracy, suggesting distinct processes underlying each measure (Segawa et al., 2019). Speech motor sequence learning is presumed to work by similar concatenation mechanisms as non-speech motor sequence learning, through which practiced speech sequences are reorganized into an optimized “chunk” (Klapp, 2003; Maas, Robin, Wright, et al., 2008; Wright et al., 2009). However, it remains unclear exactly how learning at the phonological and motor programming levels interact to result in improvements in speed vs. accuracy.

The Gradient Order Directions Into Velocities of Articulators (GODIVA) model is a neurocomputational model that specifically attempts to bridge this gap between language and motor systems (Bohland et al., 2010; Guenther, 2016). GODIVA models speech sequencing through separate phonological and motoric processes, represented by distinct nodes in the model: a phonological content buffer in the left posterior inferior frontal sulcus (pIFS), in which upcoming phonological units are temporarily stored in the proper order, and a speech sound map in the left ventral premotor cortex (vPMC) containing motor programs for frequently produced sequences.

Functional neuroimaging studies support this account. Specifically, learning non-native clusters results in decreased neural activation in the left pIFS and vPMC compared to production of untrained non-native consonant clusters (Masapollo et al., 2021; Segawa et al., 2015). Within the GODIVA framework, accuracy improvements for trained clusters result from increased processing efficiency as the result of this concatenation (either at the level of the phonological content buffer and/or the speech sound map). Duration decreases for trained clusters would be explained as either due to (i) reduced length of the sequence stored in phonological working memory, specifically in the left pIFS, resulting in faster read-out times (Klapp, 1995; Sternberg et al., 1978), and/or (ii) optimization of the motor program for this syllable. Prior behavioral work suggests that improvements in duration may reflect concatenation at the phonological level (i.e., a cluster-sized representation as part of an onset–nucleus–coda syllable frame structure; Shattuck-Hufnagel, 1992), while improvements in accuracy result from concatenation of the practiced syllable as a single motor program (Segawa et al., 2019).

Against this background, this study investigates the behavioral and neural correlates of speech motor learning in a cohort of patients with primary progressive aphasia (PPA) with the aim of dissociating the impacts of brain atrophy in key phonological and speech motor regions on learning outcomes. PPA is a neurodegenerative syndrome characterized by the presence of frontotemporal brain atrophy resulting in a disruption in speech-language abilities as the prominent and initial presenting symptom (Mesulam, 2003), typically presenting as a primary impairments in semantics (semantic variant PPA), phonology (logopenic variant PPA), or grammar and/or motor speech (nonfluent variant PPA; Gorno-Tempini et al., 2011). The study of brain–behavior correspondence in PPA provides a complementary approach to corroborate and extend poststroke lesion–symptom mapping. Atrophy in PPA follows functional speech-language brain networks with a close brain–behavior correspondence (Rogalski et al., 2011), allowing for decoupling of brain damage from brain vasculature (Mandelli et al., 2016; Seeley et al., 2009). The heterogeneity in speech-language abilities across PPA variants (Gorno-Tempini et al., 2011), in combination with the spatial resolution and sensitivity of cortical thickness measures, allows for highly reliable brain–behavior correlation analyses (Dickerson et al., 2008). For example, Rogalski and colleagues demonstrated that atrophy patterns were more strongly associated with performance in specific language domains than with a particular PPA variant; therefore, structure–function relationships were more robust when measured across PPA variants to capitalize on the variability in participants’ clinical presentation (Rogalski et al., 2011). This approach has been successfully implemented to investigate the neural bases of numerous speech-language processes in PPA (e.g., Collins et al., 2017; Cordella et al., 2019; Rogalski et al., 2011).

Furthermore, the identification of behavioral and neural predictors of speech learning in PPA is a critical step toward ultimately improving clinical recommendations for this population. Speech-language therapy in this population is relatively understudied, and there is a paucity of evidence for treatments targeting speech in particular (Wauters et al., 2024). Neurostimulation, in combination with behavioral speech-language therapy, is one promising avenue; however, it remains unclear what clinical and neural factors influence heterogeneity in intervention outcomes or how to optimize learning outcomes in PPA (e.g., Nissim et al., 2022; Tsapkini et al., 2018).

Our first research question analyzes the relationship between speech learning outcomes on the non-native consonant cluster task and clinical measures of phonological working memory and motor programming skill. We further hypothesize that (1) phonological skill will predict improvements in speed while (2) motor programming skill will predict improvements in accuracy based on prior behavioral results (Segawa et al., 2019).

Our second research question investigates neural predictors of speech motor learning. Specifically, we hypothesize that the left pIFS and left vPMC are key cortical areas for speech motor sequencing and should be correlated with baseline performance and non-native consonant cluster learning. We further hypothesize that (1) reductions in duration will be more tightly correlated with cortical thickness in the left pIFS and that (2) error rate reductions will be more strongly associated with cortical thickness in the left vPMC. Correlations between the left pIFS and improved speed would suggest duration changes are driven primarily by decreased working memory load from the phonological content buffer, while the opposite finding—reduced duration correlated with the left vPMC cortical thickness—would instead implicate motor programming optimization. Significant effects across both regions would support a combined phonological and motor learning mechanism in duration learning. Similarly, error rate reductions with either the left pIFS or the left vPMC would implicate phonological or motor learning processes, respectively.

## METHODS

### Participants

Thirty adults with PPA were recruited for this study from an existing longitudinal cohort. All patients in the cohort receive a standard clinical evaluation comprising a structured history obtained from both the patient and the informant; comprehensive medical, neurological, and psychiatric history and exams; neuropsychological and speech-language assessments; and a clinical brain MRI that was visually inspected for (1) regional atrophy consistent with or not consistent with a given syndromic diagnosis and (2) other focal brain lesions or evidence of cerebrovascular disease. Clinical formulation is performed through a consensus conference by a multidisciplinary team of neurologists, neuropsychologists, and speech-language pathologists, with each patient classified based on all available clinical information as having mild cognitive impairment or dementia (cognitive functional status), cognitive-behavioral syndrome, and likely etiologic diagnosis (Dickerson et al., 2017), following standard diagnostic criteria (Gorno-Tempini et al., 2011). Each participant in this cohort and their informant give written informed consent in accordance with human subjects research guidelines.

Participants in the present study had no history of other neurological disorders and were all native American English speakers with no prior experience with any of the languages used in our experimental stimuli. Four participants were removed due to technical issues impacting recording quality and/or precluding completion of experiment (*n* = 2) or self-withdrawal from study (*n* = 2). An a priori power analysis indicated a sample size of 28 participants would provide sufficient power (85%) to detect brain–behavior correlations of the same size as those observed in a prior reference study (*R*^2^ = 0.20; Miller et al., 2021) using multiple regression and including up to four control covariates. An additional two participants were enrolled to allow for expected attrition for acquisition of neuroimaging data in particular. The study was approved by the Boston University Institutional Review Board, and informed consent was obtained prior to participation.

Participants had a mean age of 69.5 years (STD = 9.2) and included 15 males. Participants were a mean of 4.3 years post-symptom onset (STD = 2.0). Our cohort included all three PPA variants, as well as three participants classified as PPA-Other who did not neatly fit the diagnostic criteria for any variant (see Table 1).

**Table 1.** Demographic and speech-language characteristics by diagnostic group, for nonfluent variant primary progressive aphasia (nfvPPA), semantic variant (svPPA), logopenic variant (lvPPA), and PPA-Other.

	**nfvPPA (*n* = 8)**	**svPPA (*n* = 6)**	**lvPPA (*n* = 9)**	**PPA-Other (*n* = 3)**
Age in years (*SD*)	70.1 (8.2)	63.1 (5.4)	71.3 (11.3)	75.3 (7.2)
Male, *n* (%)	4 (50)	5 (83)	5 (56)	1 (33)
Right-handed, *n* (%)	6 (75)	6 (86)	8 (89)	3 (100)
PASS-SB score (*SD*)	3.6 (2.9)	4.7 (4.3)	7.0 (2.8)^a^	2.3 (1.0)
Education in years (*SD*)	15.5 (2.8)	16.8 (2.9)	18.2 (1.6)	17 (4.6)
Time from onset in years (*SD*)	2.8 (0.9)	6 (2.4)	4.4 (1.7)	4.3 (2.1)

*Note*. PASS-SB = Progressive Aphasia Severity Score, Sum of Boxes; *SD* = standard deviation.

^a^ Incomplete data (missing 1 lvPPA).

### Experimental Setup

Data were collected remotely via Zoom videoconferencing software. To maximize recording quality, the following steps were attempted with all participants: (a) shipping high-quality recording equipment to participants (circumaural Koss UR20 headphones and Fifine K031B microphone); (b) enabling “original sound” in Zoom to disable supplemental processing and ensure the highest recording fidelity; and (c) real-time recording of the microphone signal directly to the experimenter’s computer through the Stereo Mix setting, which allows for recording from telepractice platforms at a high sampling frequency in an uncompressed, high-quality format (Weerathunge et al., 2021). Given the variation in participants’ technical expertise, language and cognitive status, and availability of caregiver support, not all measures could be successfully implemented for all participants. Any participants with recording issues that precluded complete data collection were excluded, as described above.

### Speech-Language Assessment Measures

An abbreviated motor speech assessment and the Digit Span Forward subtest from the Uniform Data Set test battery (Besser et al., 2018; Weintraub et al., 2009) were administered by the first author to obtain the primary clinical measures of motor programming skill (sequential motion rate [SMR]) and phonological skill (digit span). SMR is clinically discriminative for motor programming impairment (Melle & Gallego, 2012; Rowe et al., 2022; Strand et al., 2014; Thoonen et al., 1999), while digit span is highly selective for phonological working memory regions, including the left pIFS (Miller et al., 2021). Patients also completed comprehensive speech-language testing with speech-language pathologists within 6 months of participation in this study (mean = 52.9 days, *SD* = 34.2). The Progressive Aphasia Severity Scale (PASS; Sapolsky et al., 2014) was also completed within 9 months of participation (mean = 72.1 days, *SD* = 67.4). Clinical measures are listed in Table 2, with further details in Supplementary Material S1 (Supporting Information can be found at https://doi.org/10.1162/NOL.a.281).

**Table 2.** Complete list of clinical measures, where *n* indicates the number of participants with data available for each clinical measure.

**Domain**	**Measure**	***n***
Motor speech	Sequential motion rate task: acoustic measures of rate, consistency, coordination (Rowe et al., 2022)	23
	Sequential motion rate task: accuracy (percent consonants correct)	26
	Alternating motion rate task (/pa/): acoustic measures of rate, consistency, coordination (Rowe et al., 2022)	25
	Alternating motion rate task: accuracy (percent consonants correct)	26
	Apraxia Battery for Adults–Second Edition, Words of Increasing Length A subtest (Dabul, 2000)	26
	Pairwise variability index (Ballard et al., 2016)	26
	PASS, Articulation rating (Sapolsky et al., 2014)	25
	PASS, Fluency rating (Sapolsky et al., 2014)	25
Repetition	Uniform Data Set Version 3, Digit Span Forward subtest (Besser et al., 2018)	26
	WAB-R, Repetition subtest (Kertesz, 2007)	26
	PASS, Repetition rating (Sapolsky et al., 2014)	25
Syntax	Northwestern Assessment of Verbs & Sentences, Sentence Comprehension subtest (Thompson, 2011)	25
Semantics	Boston Naming Test, 30-item version from the Uniform Data Set Version 3 (Besser et al., 2018; Kaplan et al., 1983)	25
	Cambridge Semantic Battery, Word–Picture Matching subtest (first 32 items only; Adlam et al., 2010)	25
	Controlled Oral Word Association Test, Category Fluency (Spreen & Strauss, 1991)	25
	Controlled Oral Word Association Test, Letter Fluency (Spreen & Strauss, 1991)	23
Written language	Boston Diagnostic Aphasia Examination, Spelling subtest (Goodglass et al., 2001)	24
	WAB-R, Comprehension of Written Sentences subtest (Kertesz, 2007)	21
Global Severity	PASS, Sum of Boxes (Sapolsky et al., 2014)	25

*Note*. PASS = Progressive Aphasia Severity Scale; WAB-R = Western Aphasia Battery–Revised.

### Experimental Protocol

Participants completed a non-native cluster learning protocol, which involved repeated productions of CCVC (C = consonant, V = vowel) pseudowords where the initial cluster is impermissible in English (e.g., “*vzat*”; Segawa et al., 2015, 2019). The protocol consisted of three phases—familiarization, training, and test—across two experimental sessions on consecutive days. The protocol, based on Segawa et al. (2019), was modified to account for the older age and anticipated speech-language difficulties in this PPA cohort. Changes to the paradigm included the following: use of simpler CCVC syllables (compared to CCVCC previously), fewer total trained pseudowords (to allow for more repetitions of each token), fewer overall trials, and shorter blocks of 45 trials to reduce potential fatigue. In all protocol phases, an auditory model of the target word was presented to participants while the orthographic visual was presented simultaneously on the computer screen. Participants were instructed to repeat each word as quickly and accurately as possible. Stimuli were presented in pseudorandom order. Productions were recorded directly to the experimenter’s computer at 48.0 kHz via Matlab.

In the *familiarization phase*, participants completed eight trials during which they received specific feedback on their productions (similar to Buchwald et al., 2019; Masapollo et al., 2021). The stimuli used in the familiarization phase (“*fshek*,” “*dzek*”) did not appear in any other protocol phase.

The *training phase* included four blocks of 45 trials on the first day and an additional two blocks of 45 trials during the second session. Each participant practiced three pseudowords, with the training list counterbalanced across participants. No feedback was provided during the training phase, except for summary feedback between blocks only in the case of a consistent, egregious error (e.g., participant always omits one sound).

On the second day, following a 15-min break after the last training block, participants completed 90 trials during the *test phase*. The test trials consisted of the three trained pseudowords intermixed with a set of nine novel pseudowords containing untrained consonant clusters. The test phase was divided into two blocks of 45 trials each with no feedback provided.

#### Speech stimuli

Experimental stimuli for the learning task consisted of four sets of CCVC pseudowords, which were randomized across participants (see Supplementary Material S2 for a complete list). Each set contained three training stimuli and nine novel test stimuli. Each stimulus contained a unique non-native syllable-initial consonant cluster from a variety of non-English languages (e.g., Polish, Russian, Hindi).

Stimuli were recorded by a trained female native speaker of American English. Tokens were heavily practiced to ensure the speaker could produce each stimulus with appropriate speed and without epenthesis. Speech was recorded directly to a computer using Audacity software in a sound-attenuated booth via a lapel microphone (Shure, SM93) connected to a pre-amplifier (Behringer Eurorack UB802; 44.1-kHz sampling rate). Multiple repetitions of each token were recorded from which one model instance was selected following review by two trained speech-language pathologists. All tokens were matched for peak intensity using Praat software (Boersma & Weenink, 2020) and for duration (i.e., 380 ms) using Audacity (e.g., Segawa et al., 2015).

### Data Preprocessing and Experimental Measures

Perceptual accuracy was scored for each trial by a phonetically trained research assistant using acoustic visualization of the waveform and spectrogram to support auditory judgments of (1) gross disfluencies, (2) productions unrecognizable from target, and (3) phoneme deletion, insertion, substitution, or transposition. For each accurately sequenced trial (i.e., accurate other than the potential presence of epenthesis), the same research assistant manually measured duration from the initial consonant onset to the end of the vowel using custom scripts in Praat software (Boersma & Weenink, 2020). Epenthesis errors were scored separately (as described by Segawa et al., 2019). However, given the focus of the GODIVA model on speech sequencing, our primary analyses focused only on sequencing errors, consistent with the framework applied in prior work (Masapollo et al., 2023; Segawa et al., 2019), where epenthesis errors are considered to be errors in articulator timing and coordination, resulting in a truncated vocoid mid-cluster despite accurate sequencing of the intended phonemes (Buchwald & Cheng, 2023; Cheng et al., 2024; Davidson, 2006).

The scorer was blind to stimulus categorization (trained vs. novel) or patient diagnosis. Approximately 20% of trials were re-scored for calculation of intrarater reliability, which was excellent for both duration (ICC(2, 1) 0.9994 [0.9994, 0.9995], < 0.001, absolute agreement on single measures) and error rate measures (ICC(2, 1) 0.989 [0.988, 0.990], < 0.001, absolute agreement on single measures).

For each participant, we calculated two test-phase learning measures: (1) percent error rate of novel stimuli (first five productions only of each stimulus, to minimize learning) minus percent error rate of trained stimuli and (2) duration of accurately sequenced novel productions (first five productions only of each stimulus) minus duration of accurately sequenced trained productions. Novel error rate was calculated as the mean across the error rates for each of the nine novel stimuli, while trained error rate was similarly calculated as the mean across each of the three trained stimuli. A duration learning measure was not calculated if participants had no accurate trials in either of the two stimulus conditions (*n* = 4).

Training-phase improvements were separately characterized through calculation of (1) percent error rate across the first 15 productions of each trained stimulus (45 trials total) minus percent error rate across the last 15 productions of each trained stimulus and (2) duration of accurately sequenced productions for the first 15 productions of each trained stimulus compared to the last 15 productions. Again, a duration learning measure was not calculated for participants with no accurate trials at either timepoint (*n* = 3). All learning measures are expected to be positive (i.e., fewer errors and shorter duration of trained items).

### MRI Cortical Thickness Measures

Neuroimaging data were collected for a subset of the cohort (*n* = 19) within a mean of 96.1 days of the behavioral session (*SD* = 104.0, range: 10–399). See Supplementary Material S3 for demographic information for the imaging cohort, summarized in Supplementary Table S3, and atrophy patterns by diagnosis in Supplementary Figure S1.

High-resolution T1-weighted structural MRI scans were collected on a 3T scanner. Most scans (*n* = 15) were acquired on a Siemens Prisma scanner with a 64-channel head coil using the standardized Alzheimer’s Disease Neuroimaging Initiative-3 three-dimensional T1 magnetization-prepared rapid acquisition gradient echo (MPRAGE) protocol (Jack et al., 2015, 2024), with repetition time (TR) = 2,300 ms, echo time (TE) = 2.98 ms, flip angle = 9.00°, number of sagittal slices = 208, field of view (FOV) = 240 × 256, slice thickness = 1.00 mm. Two participants were scanned on a Siemens Vida scanner with a 20-channel head coil using a similar MPRAGE protocol, but with number of sagittal slices = 176, FOV = 248 × 256, and slice thickness = 1.10 mm instead. Data for one participant were acquired on a GE SIGNA Premier scanner with a BRAVO MPR protocol with TR = 2,447.3 ms, TE = 2.8 ms, flip angle = 8.00°, number of sagittal slices = 216, FOV = 240 × 192, slice thickness = 1.00 mm.

MRI data were visually inspected for gross artifacts and image quality (Jack et al., 2015). Cortical surface reconstructions were then generated using FreeSurfer software v6.0 (Dale et al., 1999). Surface reconstructions were visually inspected by the first author, and any surface errors resulting from poor image segmentation were manually edited. Subject-specific regions of interest (ROIs) were identified using the SpeechLabel parcellation scheme (Tourville & Guenther, 2012), and average cortical thickness measures were extracted for each ROI. Within each ROI, any data points exceeding 3.5 standard deviations from the cohort mean for that ROI were removed (*n* = 1).

### Statistical Analyses

#### Covariate analyses

Preliminary analyses explored the relationship between primary experimental measures (behavioral and MRI) with potential demographic covariates. Spearman correlations conducted for potential covariates (age, sex, handedness) identified no significant relationships with any learning measures or with the two primary clinical measures (SMR, digit span score). A multiple regression analysis showed no evidence of significant covariation between potential covariates and aggregated cortical thickness across all ROIs in the parcellation.

#### Clinical correlation analyses

##### Behavioral predictors of novel speech motor sequence repetition.

Separate Spearman correlations were conducted to assess the relationship between novel speech motor sequence repetition (i.e., error rate and duration of novel pseudowords during the test phase) and (a) the a priori hypothesized clinical measures (digit span and SMR) and (b) all available clinical measures in an exploratory analysis, with false discovery rate (FDR) corrections and alpha = 0.05. Participants with missing data were removed only from the analysis for that measure.

##### Behavioral predictors of speech motor sequence learning.

Separate Spearman correlations were conducted to assess the relationship between each learning parameter (i.e., error rate and duration) and the selected clinical measures (digit span and SMR). Exploratory Spearman correlation analyses were conducted across all clinical measures, with FDR corrections applied.

#### Neuroimaging analyses

##### Neural correlates of novel speech motor sequence repetition.

Separate regression analyses were conducted between baseline performance measures (i.e., error rate and duration of novel pseudowords during the test phase) and cortical thickness measures, (a) for the a priori hypothesized ROIs (left pIFS and left vPMC) and (b) in an FDR-corrected exploratory whole-brain analysis. Analyses included age and handedness as covariates. One-tailed analyses were performed for all neuroimaging analyses due to the unidirectional hypothesis of impaired performance with cortical thinning, with alpha = 0.05. Participants with missing data (i.e., no duration learning measure) were removed from only that analysis.

##### Neural correlates of speech motor sequence learning.

Separate regression analyses were conducted to identify significant relationships between each learning parameter (i.e., duration and error rate) and cortical thickness measures for (a) the hypothesized ROIs (left pIFS and left vPMC) and (b) an FDR-corrected whole-brain exploratory analysis. Covariates included age, handedness, and baseline performance. For training-phase analyses, performance at the beginning of training was used as the baseline measure. For test-phase analyses, performance on novel items was used as the baseline metric.

## RESULTS

### Learning

Wilcoxon signed-rank tests for the full cohort confirmed significant reductions in both error rate and duration across the training phase (first 15 productions of each stimulus compared to the last 15; error rate: *Z* = 2.427, *p* = 0.015; duration: *Z* = 2.707, *p* = 0.007). Differences between trained and novel items during the test phase were significant only for duration (error rate: *Z* = 1.232, *p* = 0.22; duration: *Z* = 1.689, *p* = 0.042). Spearman correlations confirmed that error rate and duration improvements were not correlated with each other in either experimental phase (training: *p* = 0.35; test: *p* = 0.80). Although learning in the test phase was not significant at the group level, within-subject Mann–Whitney *U* tests showed significant learning for trained compared to novel tokens (i.e., test phase) for eight participants for error rate and seven participants for duration; within-subject training-phase Mann–Whitney *U* tests also showed significant learning for eight participants each for duration and error rate measures. Overall, 15 of 26 participants showed significant learning on at least one metric.

Within imaging participants, significant changes across the training phase were observed only for error rate (*Z* = 2.102, *p* = 0.036), while duration was marginally significant (*Z* = 1.894, *p* = 0.058). Differences between trained and novel items during the test phase were not significant (error rate: *Z* = 1.449, *p* = 0.14; duration: *Z* = 1.255, *p* = 0.21). Again, while test-phase learning measures were not significant at the group level for the imaging cohort, within-subject analyses revealed statistically significant increases in performance for some participants (seven for error rate, six for duration). Raw means for both the full cohort and the imaging subset are summarized in Table 3.

**Table 3.** Summary of mean behavioral measures and mean learning measures for both error rate and duration across the full cohort as well as within only the imaging cohort.

	**Training: start**	**Training: end**	**Learning: training**	**Test: novel**	**Test: trained**	**Learning: test**
Error rate–full cohort (%)	61.5	50.2	11.4**	52.1	48.1	4.0
Error rate–imaging (%)	60.3	46.0	14.3**	49.0	43.8	5.1
Duration–full cohort (ms)	447.3	396.8	47.7***	418.8	379.6	29.2**
Duration–imaging (ms)	416.3	382.5	33.9*	382.3	358.1	24.6

*Note*. The training learning measure for each participant is measured as performance at the start of training minus performance at the end of training, while the test learning measure is novel stimuli performance minus trained stimuli performance. Significance is indicated as follows: *p < 0.1, **p < 0.05, ***p < 0.01.

### Clinical Correlation Analyses

#### Behavioral predictors of novel speech motor sequence repetition

Spearman correlations testing the a priori hypotheses identified significant correlations between novel error rate and digit span (*r*_s_ = −0.497, *p* = 0.010), but not with SMR, and between novel duration and SMR (*r*_s_ = −0.502, *p* = 0.032), but not with digit span.

Spearman analyses across all available clinical measures identified additional significant correlations between novel error rate and performance on the Western Aphasia Battery (WAB) Repetition subtest (*r*_s_ = −0.670, *p* = 0.0002, *p*-FDR = 0.004), the PASS Sum of Boxes clinician rating (*r*_s_ = 0.604, *p* = 0.001, *p*-FDR = 0.015), and the PASS Repetition clinician rating (*r*_s_ = 0.574, *p* = 0.003, *p*-FDR = 0.019). For each measure, higher performance on this clinical measure was associated with fewer errors on novel pseudowords.

Spearman correlation analyses of novel duration revealed significant correlations with alternating motion rate (AMR; *r*_s_ = −0.572, *p* = 0.004, *p*-FDR = 0.048), the Boston Naming Test (*r*_s_ = 0.555, *p* = 0.005, *p*-FDR = 0.048), and the Cambridge Semantic Battery (*r*_s_ = 0.537, *p* = 0.007, *p*-FDR = 0.043). Of these measures, AMR was the only measure where better performance was associated with shorter durations of novel items. Instead, better semantic performance was associated with longer durations.

#### Behavioral predictors of speech motor sequence learning

##### Error rate.

First, to test the hypothesis that clinical measures of motor programming ability and phonological working memory are predictive of learning, we performed Spearman correlations between the two hypothesized measures (digit span and SMR) and the error rate learning measures. Spearman correlations revealed significant correlations between the test-phase learning measure and digit span score (*r*_s_ = 0.48, *p* = 0.013), but not for SMR (*p* = 0.35). Digit span scores were also correlated with improvements in error rate across the training phase (digit span: *r*_s_ = 0.40, *p* = 0.046; SMR: *p* = 0.21).

Exploratory learning analyses conducted across all clinical measures identified significant correlations only between the test-phase error rate learning measure and the PASS Repetition rating (*r*_s_ = −0.610, *p* = 0.0011, *p*-FDR = 0.025). Participants with higher performance in that domain also demonstrated larger improvements in accuracy on trained items. No clinical measures survived correction for training-phase analyses.

##### Duration.

Spearman correlations testing the a priori hypotheses for duration (both test and training learning measures) were not significant for either SMR or digit span (*p* > 0.11). No clinical measures survived FDR correction for correlation analyses with either test or training measures of learning.

### Neuroimaging Analyses

#### Neural correlates of novel speech motor sequence repetition

##### Error rate.

To test our primary hypothesis that the left pIFS and left vPMC are key cortical areas for speech motor sequencing, we tested for correlations between cortical thickness in these two ROIs and baseline error rate (i.e., error rate on novel pseudowords). Regression analysis revealed a significant correlation with cortical thickness in the left vPMC (*β* = −0.501, *t*(15) = −2.243, *p* = 0.020), but not in the left pIFS (*p* = 0.11).

**Figure 1.**
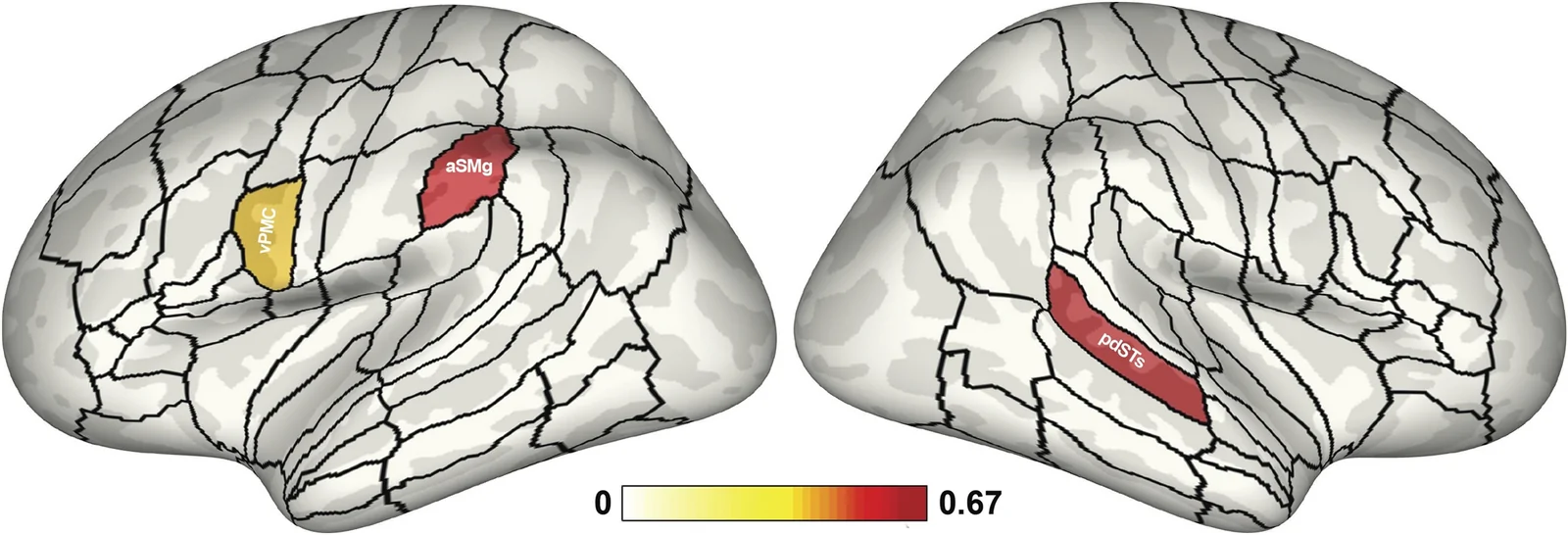
ROIs significantly correlated with error rate for novel pseudowords during the test phase. Colors represent standardized regression coefficients (*β*), with warmer colors indicating stronger associations between cortical thickness and accuracy. aSMg = anterior supramarginal gyrus; pdSTs = posterior dorsal superior temporal sulcus; vPMC = ventral premotor cortex.

Whole-brain regression analysis identified the following ROIs that are significantly correlated with novel error rate: left anterior supramarginal gyrus (aSMG; *β* = −0.712, *t*(15) = −3.923, *p-unc* = 0.00068, *p-FDR* = 0.045) and right superior temporal sulcus, posterior dorsal division (pdSTS; *β* = −0.754, *t*(15) = −4.443, *p-unc* = 0.00024, *p-FDR* = 0.031). Results are displayed in Figure 1.

##### Duration.

First, to test the a priori hypotheses, uncorrected regression analyses between baseline duration (i.e., duration of novel pseudowords during the test phase) and cortical thickness in the left pIFS and left vPMC revealed that neither ROI was significantly associated with baseline duration (*p* > 0.19).

Whole-brain exploratory analysis revealed that novel pseudoword duration was correlated only with cortical thickness in right ventral motor cortex, but this result did not survive FDR corrections (*β* = −0.487, *t*(14) = −2.088, *p* = 0.028, *p*-FDR = 0.99).

#### Neural correlates of learning

##### Error rate.

###### A priori hypothesis tests.

Our primary imaging hypothesis was that the left pIFS and left vPMC will be key neural correlates of learning outcomes. Regression analysis for the test-phase error rate learning measure (i.e., trained compared to novel) revealed a significant correlation with the left pIFS (*β* = −0.583, *t*(14) = 2.687, *p* = 0.0088), but not the left vPMC (*p* = 0.19). Improvement in error rate across the training phase was significantly correlated with only the left vPMC, although the left pIFS was borderline significant (vPMC: *β* = 0.484, *t*(14) = 2.069, *p* = 0.029; pIFS: *β* = 0.370, *t*(14) = 1.488, *p* = 0.079). Scatter plots for both analyses are available in Supplementary Material S4.

###### Whole-brain exploratory analysis.

No regions survived FDR correction for either error rate learning analysis (training phase or test phase).

This whole-brain exploratory analysis was repeated for the training-phase learning measure (i.e., improvement in error rate across the training phase), Although they did not survive corrections, the training-phase analysis revealed uncorrected correlations with both ROIs that were previously identified in the novel error rate analysis: left aSMG (*β* = 0.523, *t*(14) = 2.297, *p* = 0.019, *p*-FDR = 0.17) and right pdSTS (*β* = 0.652, *t*(14) = 3.220, *p* = 0.003, *p*-FDR = 0.17). Given the small sample size and the exploratory nature of this work, these uncorrected results are presented here as candidates for future exploration in larger cohorts.

##### Duration.

###### A priori hypothesis tests.

Regression analyses were performed to test the a priori hypothesis regarding the role of the left vPMC and left pIFS in learning, with no significant correlations identified between the test-phase duration learning measure and either the left vPMC or left pIFS (*p* > 0.24). Improvement in duration across the training phase was significantly correlated with the left vPMC (*β* = 0.672, *t*(13) = 3.275, *p* = 0.003), but not the left pIFS (*p* = 0.35). Scatter plots for each ROI are available in Supplementary Material S4.

###### Whole-brain exploratory analysis.

Whole-brain analysis for the test-phase duration measure revealed correlations with cortical thickness in the right lingual gyrus (*β* = 0.839, *t*(12) = 5.343, *p* < 0.0001, *p*-FDR = 0.012).

**Figure 2.**
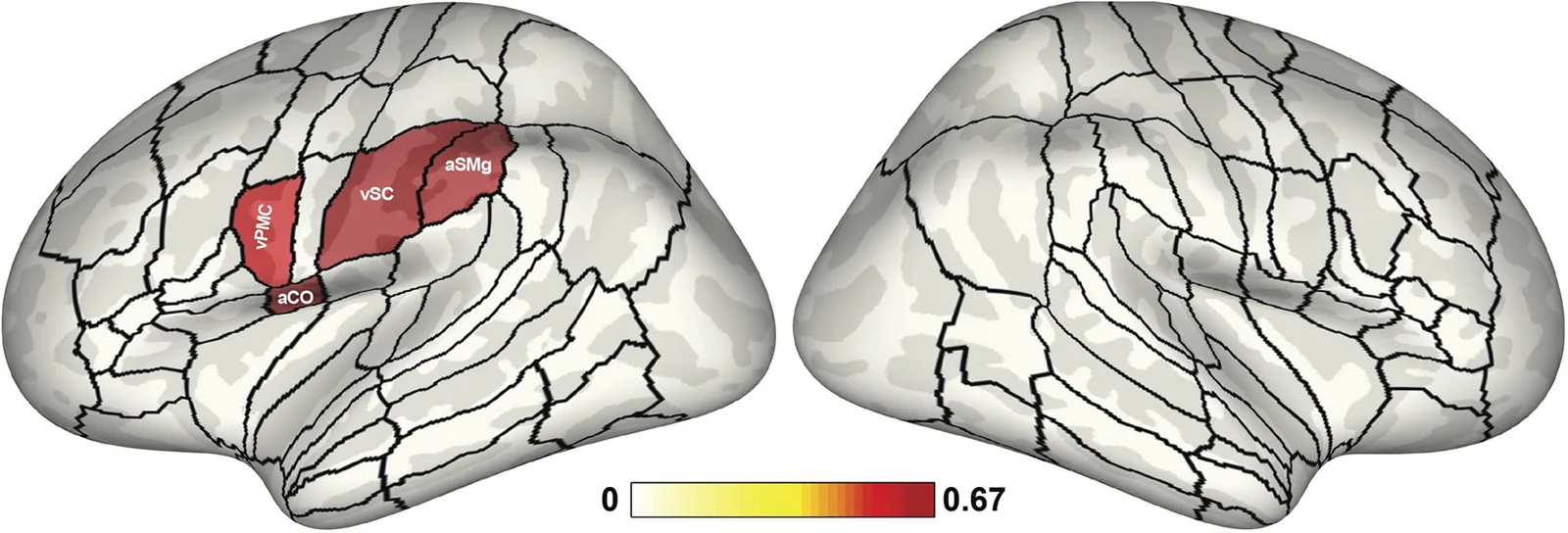
ROIs significantly correlated with learning results from training-phase duration learning analyses. Colors represent standardized regression coefficients (*β*), with warmer colors representing stronger associations between cortical thickness and learning. aCO = anterior central operculum; aSMg = anterior supramarginal gyrus; vPMC = ventral premotor cortex; vSC = ventral somatosensory cortex.

Whole-brain analysis was repeated for the training-phase measure, revealing correlations with the following ROIs: left aSMG (*β* = 0.789, *t*(13) = 4.625, *p* = 0.0002, *p*-FDR = 0.020), left ventral somatosensory cortex (*β* = 0.781, *t*(13) = 4.506, *p* = 0.0003, *p*-FDR = 0.020), and left anterior central operculum (*β* = 0.756, *t*(13) = 4.162, *p* = 0.0006, *p*-FDR = 0.025). Results are summarized in Figure 2.

## DISCUSSION

This study aimed to determine clinical and neural correlates of speech motor sequence learning in PPA. Participants improved in both accuracy and speed of trained non-native consonant clusters, particularly across the training phase. Results partially supported the primary hypothesis that the left pIFS and left vPMC are key cortical areas for speech motor sequencing and learning. However, the left pIFS findings were specific to improvements in accuracy, opposite to our secondary hypothesis that duration would be more strongly correlated with thickness in the left pIFS. Atrophy in the left vPMC was associated with worse learning outcomes for both speed and accuracy, but only across the training phase. In other words, individuals with thicker cortex in vPMC showed larger gains during training, but their improvements were not specific to the trained words. Exploratory analyses identified distinct clinical correlates of speed and accuracy: Duration was best predicted by clinical motor speech measures, while error rate was associated with clinical repetition tasks. Neuroimaging analyses identified associations between the left aSMG and both duration and error rate, while premotor and sensorimotor regions were distinct to duration improvements and the right pdSTS was unique to error rate.

### Clinical Predictors of Speech Motor Sequence Learning

Error rate analyses identified two clinical measures that were predictive of both baseline error rate and learning (specifically test phase): digit span and the PASS repetition clinician rating. Digit span and other verbal repetition tasks are used clinically to gauge the integrity of phonological working memory mechanisms (e.g., Gorno-Tempini et al., 2008; Miller et al., 2021). The PASS Sum of Boxes score was uniquely predictive of baseline error rate, suggesting that a global progression in disease severity may impair repetition accuracy beyond the impact of phonological working memory ability alone.

In contrast, baseline duration on novel pseudowords was predicted by clinical motor speech measures, AMR and SMR rate, suggesting that the presence of motor speech impairment was the strongest factor in baseline speech rate (Duffy, 2013; Melle & Gallego, 2012). A secondary contributor to baseline duration was a cluster of semantic measures—the Boston Naming Test and the Cambridge Semantic Battery—that were inversely correlated with speed. Essentially, the presence of semantic impairment was indicative of preserved motor speech abilities, consistent with a review that identified only a single case of motor speech disorder across semantic PPA patients from 12 published cohorts (Duffy et al., 2014).

Consistent with a priori hypotheses, both forward digit span and SMR were significant predictors of baseline speech motor sequencing performance. However, contrary to hypotheses, SMR was a predictor of baseline duration, while digit span was correlated with baseline error rate and learning. In combination, these findings suggest that duration is driven more by motor programming mechanisms than phonological skill. Underlying phonological working memory ability instead contributes more strongly to accuracy.

### Neural Correlates of Speech Motor Sequence Learning

#### Error rate

Our a priori imaging hypotheses tested the proposed function of the left pIFS and left vPMC within the speech sequencing network. Findings were somewhat inconsistent across analyses but broadly supported the hypothesized involvement of these ROIs in speech sequencing and learning.

**Figure 3.**
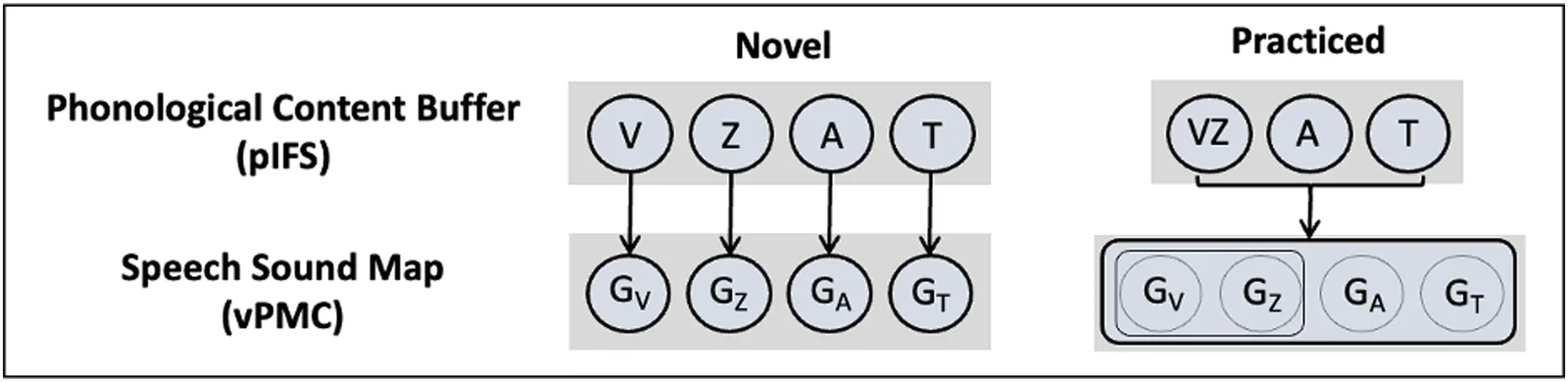
Schematic of the GODIVA model account of speech motor sequence learning, comparing a novel sequence and a practiced sequence. GODIVA describes two distinct learning processes that occur at the phonological and motoric levels. G_X_ = articulatory gesture for phoneme x.

Although hypothesized to be more closely tied to duration, the left pIFS was instead correlated with improvements in error rate in both the test phase and (marginally significant) training phase. Interpreted within the GODIVA model (see Figure 3), this result suggests patients with thicker cortex in the left pIFS may be better able to acquire a new representation for the trained cluster as a single unit in the phonological content buffer. However, this chunking mechanism appears to exclusively improve accuracy of these trained clusters (as opposed to duration), particularly when compared to novel items, which are represented as single phoneme-sized units within the left pIFS and would therefore be more prone to sequencing errors.

Our findings seem at odds with prior behavioral findings of improvements in speed as a result of chunking specifically at the cluster level, which would suggest pIFS involvement (Segawa et al., 2019). Segawa and colleagues postulated that the reduced sequencing load for trained pseudowords resulted in faster performance on these items. Based on our findings, it appears that chunking at the phonological level (presumed to take place in the left pIFS, per the GODIVA model) may instead have a greater impact on accuracy. More recently, transcranial direct current stimulation (tDCS) applied to the left pIFS during non-native cluster learning has been shown to improve accuracy only, and not duration (Rowe et al., 2025), in keeping with the current findings.

Consistent with our hypothesis, the left vPMC was correlated with both baseline error rate on novel pseudowords and improvements in error rate across the training phase. However, we did not observe the predicted dissociation between error rate and duration in this ROI; instead, the left vPMC was also associated with improvements in duration across the training phase. According to the GODIVA model, the left vPMC atrophy may correspond with either impairments in motor program selection (resulting in activation of the wrong movement gesture) or reduced integrity of motor programs (resulting in imprecise movements). Both mechanisms would be expected to lead to a higher error rate on novel pseudowords. We hypothesized that speakers with less atrophy in the left vPMC would be better able to consolidate the learned pseudowords into a novel motor program, resulting in fewer errors following practice. Prior neuroimaging studies show reduced activation in both the left pIFS and left vPMC following learning (trained-novel contrast), consistent with fewer units that must be activated following this concatenation process (Masapollo et al., 2021; Segawa et al., 2015).

Whole-brain analysis of baseline error rate identified significant correlations with cortical thickness in the left aSMG and right pdSTS. Both regions play a role in phonological processing. The left aSMG is implicated in phonological working memory as an input buffer that works in concert with a phonological output buffer in the left pIFS (Jacquemot & Scott, 2006; Miller et al., 2021), while the right pdSTS is important for speech perception (Hamilton et al., 2018; Hickok & Poeppel, 2007). These regions can be viewed as linking acoustic representations to phonological representations. Both ROIs also feature prominently in the DIVA model of speech production, a partner model to GODIVA that describes lower-level speech motor control (Golfinopoulos et al., 2011; Guenther, 2016; Tourville et al., 2008). The left aSMG and right pdSTS form part of larger somatosensory and auditory maps, respectively, where the current sensory state is compared to a predicted sensory target to generate an error signal in the case of a mismatch (Guenther, 2016). In combination, these results suggest preserved phonological working memory skills and/or feedback control systems allow for more accurate repetition of non-native pseudowords. Of note, both ROIs were also correlated with improvements in error rate across the training phase, but this finding did not survive FDR corrections for multiple corrections. Nonetheless, these ROIs appear to play a complementary role to the hypothesized GODIVA regions that warrants further exploration.

#### Duration

Training-phase duration improvements were most strongly correlated with the left aSMG and neighboring ventral somatosensory cortex. These regions are thought to process somatosensory feedback during speech production, which is necessary to refine the timing of speech gestures for faster productions (Golfinopoulos et al., 2011; Guenther, 2016). The left aSMG was also predictive of baseline error rate and (uncorrected) correlations with training-phase improvements in error rate. Although our results largely showed a dissociation between duration and error rate, the left aSMG was notable for correlations with both outcome measures, perhaps reflecting the contributions of both phonological and motor functions within this region.

Training-phase duration improvements were also correlated with the left vPMC and neighboring central operculum, which reflect feedforward contributions to support acquisition of a novel motor program (Guenther, 2016). According to the GODIVA model, individual motor programs in the left vPMC are consolidated into an optimized motor program to allow for coordinated and automatic production of the entire syllable following learning (see Figure 3). Again, we did not observe the predicted dissociation between error rate and duration for this ROI. Within the GODIVA framework, correlations with the left vPMC indicate that concatenation of smaller motor gestures into a larger motor program allows for both faster and more accurate transitions between sounds. However, this interpretation is tempered by the finding that cortical thickness in the left vPMC did not predict test-phase learning measures. In current computer instantiations of the DIVA model, the learning of a motor program for one syllable does not generalize to other syllables; our finding of only training-phase correlations with vPMC thickness suggests that motor program learning may involve both word-specific and generalizable components.

In contrast, the strongest test-phase correlations with duration were centered around the right lingual gyrus, which is associated with visual memory and sensitive to global word shape (Bogousslavsky et al., 1987; Fink et al., 1996). One important caveat, however, is that group-level learning analyses for the imaging cohort revealed no significant learning (trained-novel contrast) during the test phase. Many participants had relatively fast durations for the novel items: A quarter of the imaging cohort produced novel pseudowords with comparable speed as the model, resulting in a ceiling effect for the test-phase learning measure. Motor speech diagnosis was comparatively underrepresented in the imaging analyses (*n* = 3, compared to *n* = 7 for behavioral analyses), which likely limited the sensitivity of the test-phase duration analyses.

### Limitations and Future Directions

It is important to note several limitations to this work. First, our assessment of learning was relatively narrow and did not probe certain key aspects of learning such as generalization and retention. Our training protocol incorporated principles that serve to maximize learning (Maas, Robin, Austermann Hula, et al., 2008), such as a prepractice period (i.e., the familiarization phase), reduced feedback, and randomized, high-intensity practice; these principles should promote generalization and retention of the practiced speech sequences. Nonetheless, as one of the first studies to investigate speech learning in PPA, these results demonstrate retained capacity for speech learning in at least some patients, consistent with findings in perceptual learning of speech (Hardy et al., 2018). Further characterization of the individual factors that support preserved learning in individuals with PPA remains an important avenue for future work to inform clinical treatment research in this population.

An additional limitation of this work is the relatively small sample size, particularly given the heterogeneity of our cohort. As a result, the sample size within each included diagnostic group is small, precluding meaningful analysis of variant-specific performance. Future work should include larger sample sizes to better characterize differences in speech learning performance across PPA variants in order to inform potential treatment options for patients with speech deficits in particular. Nonetheless, prior work has established a clear benefit to investigating brain–behavior correspondence in heterogeneous dementia cohorts, rather than studying a single diagnosis in isolation. For example, Rogalski and colleagues demonstrated that patients with PPA frequently present with secondary deficits outside of the primary impacted domain, with a close correspondence between these secondary deficits and atrophy in associated cortex; therefore, structure–function relationships were more robust when measured across PPA variants to capitalize on this variability (Rogalski et al., 2011).

Speech-language therapy for PPA, especially progressive apraxia of speech, has historically featured lower rates of direct speech-language intervention when compared to management of stroke-based aphasia or apraxia, with limited research on the efficacy of direct speech intervention in this population (Duffy et al., 2021; Wauters et al., 2024). This study is therefore a necessary contribution to the emerging evidence base investigating learning in PPA. With the advent of potential adjunctive targeted interventions in PPA, characterization of the mechanisms underlying speech-language learning will allow for more effective interventions that target these neural bases. Patient-specific atrophy data in PPA may be able to predict learning prognosis; for example, Nissim and colleagues found that baseline cortical thickness measures predicted response to tDCS treatment in PPA (Nissim et al., 2022).

Behavioral data for this study were collected remotely via videoconferencing software to increase the accessibility of this work for a difficult-to-recruit population. This decision allowed us to include a more diverse sample of patients in the study, including many who were physically unable to travel into our laboratory for consecutive in-person visits. Evidence supporting the validity of videoconferencing software use continues to emerge, including for acoustic measurement of duration or rate (Conklin, 2023; Python et al., 2023), perceptual evaluation of speech-sound accuracy (Bartamay et al., 2023), and in PPA and dementia more broadly (Weidner & Lowman, 2020). The growing use of videoconferencing software in both research and clinical practice has the potential to improve accessibility of research for larger and more representative research samples, as well as to increase the availability of behavioral speech-language intervention for patients with PPA.

Lastly, this work does not account for the contribution of subcortical and cerebellar structures in speech sequence learning. The GODIVA model incorporates a basal ganglia-thalamo-cortical loop that executes speech sequences, in concert with the above described premotor regions (Bohland et al., 2010). Prior non-native cluster learning studies in neurotypical adults have shown changes in functional activation in both the basal ganglia (Segawa et al., 2015) and cerebellum (Masapollo et al., 2021). Anodal tDCS stimulation to the right cerebellum has also been shown to improve non-native cluster learning outcomes (Rowe et al., 2025). The application of complementary neuroimaging techniques, including functional neuroimaging, will allow for a more complete characterization of the dynamic nature of speech sequence learning in future work.

## ACKNOWLEDGMENTS

The authors are grateful to Hannah Indiviglio for her assistance with behavioral data scoring and acoustic analysis, and to Nneka Watson, Megan Quimby, and Sophia Tchir-Bourgeois for their contributions to our recruitment efforts. Finally, we are grateful to our patients and their families for volunteering their time to participate in this research.

## FUNDING INFORMATION

Hilary E. Miller, National Institute on Deafness and Other Communication Disorders (https://dx.doi.org/10.13039/100000055), Award ID: F31 DC020352. Frank H. Guenther, National Institute on Deafness and Other Communication Disorders (https://dx.doi.org/10.13039/100000055), Award ID: R01 DC007683. Bradford C. Dickerson, National Institute on Deafness and Other Communication Disorders (https://dx.doi.org/10.13039/100000055), Award ID: R01 DC014296. Bradford C. Dickerson, National Institute of Neurological Disorders and Stroke (https://dx.doi.org/10.13039/100000065), Award ID: R01 NS131395. Hilary E. Miller, Acoustical Society of America (https://dx.doi.org/10.13039/100005299), Award ID: Raymond J. Stetson Scholarship.

## AUTHOR CONTRIBUTIONS

**Hilary E. Miller**: Data curation: Lead; Formal analysis: Lead; Funding acquisition: Equal; Investigation: Lead; Methodology: Equal; Project administration: Lead; Software: Equal; Visualization: Lead; Writing – original draft: Lead. **Michael Brickhouse**: Data curation: Supporting; Formal analysis: Supporting; Writing – review & editing: Supporting. **Daisy Hochberg**: Data curation: Supporting; Resources: Supporting; Writing – review & editing: Supporting. **Jason Tourville**: Methodology: Equal; Visualization: Supporting; Writing – review & editing: Supporting. **Alfonso Nieto-Castanon**: Methodology: Equal; Software: Equal; Visualization: Supporting; Writing – review & editing: Supporting. **Bradford C. Dickerson**: Conceptualization: Equal; Funding acquisition: Equal; Methodology: Equal; Resources: Equal; Supervision: Equal; Writing – review & editing: Supporting. **Frank H. Guenther**: Conceptualization: Equal; Funding acquisition: Equal; Methodology: Equal; Resources: Equal; Supervision: Equal; Writing – review & editing: Lead.

## DATA AND CODE AVAILABILITY STATEMENTS

The code supporting the findings of this study is available on GitHub at https://github.com/HEMiller/NOL2026. The data supporting the findings of this study are available on OSF at https://osf.io/p2xuc.

## Supplementary Material



## TECHNICAL TERMS

Speech motor sequence learning: The process by which speakers learn to plan and execute an unfamiliar combination of phonemes and/or syllables in the correct order.
Epenthesis: Insertion of a vocoid, often between two consonants in a cluster.
