# Supplementary material for: Neural and Behavioral Predictors of Speech Motor Sequence Learning in Primary Progressive Aphasia
Source: Neurobiol Lang (Camb). 2026 Sep 4;7:NOL.a.281. doi: 10.1162/NOL.a.281 (PMC13612843; doi:10.1162/NOL.a.281)

Supplementary Materials 1: Speech-language assessment measures.

Motor speech measures.

An abbreviated motor speech assessment was completed during the experimental sessions, with high-quality recordings obtained for diadochokinetic tasks, multisyllabic word production, and the Words of Increasing Length A subtest from the Apraxia Battery for Adults-Second Edition (Dabul, 2000). Scoring for the Words of Increasing Length A subtest was modified from that described in the manual to avoid potential nonlinearity effects, instead following the formula used by Ballard and colleagues (2016): ((Column 1 score – Column 3 score) + 1) / (Column 1 score + 1), where Column 1 indicates the total score on monosyllable words, while Column 3 indicates the total score on all three syllable words.

Diadochokinetic tasks included both alternating motion rate (AMR) and sequential motion rate (SMR). The primary measure extracted to index motor programming skill for the hypothesis-driven analysis was repetition rate during the SMR task, since this measure is particularly discriminative for motor programming impairment (Melle & Gallego, 2012; Rowe et al., 2022; Strand et al., 2014; Thoonen et al., 1999). Participants were instructed to produce each sequence as quickly and accurately as possible until they ran out of breath. Participants were allowed multiple attempts, but rate was calculated only for each participant's first production that was correct at the articulatory placement level (i.e., ignoring voicing errors or distortion errors) across the first nine syllables, consistent with the criteria utilized by Ballard and colleagues (2016). SMR could only be calculated for 23/26 participants; the remaining three participants were unable to produce three accurate repetitions of /pataka/ despite multiple attempts.

Rate for both SMR and AMR tasks was measured consistent with the methods utilized by Rowe and colleagues (2022). For each participant, duration of the first nine syllables (measured from the plosive in syllable one through to plosive marking the beginning of syllable ten) was measured by a trained research assistant, who re-measured data from approximately 20% of participants to establish intra-rater reliability. Intraclass correlation coefficients confirmed excellent intra-rater reliability (ICC(2,1) .999 [.997, .9995], < .001, absolute agreement on single measures), with an average difference of 9.0 ms (SD = 18.1 ms).

Diadochokinetic analyses also included Consistency and Coordination measures, as defined by Rowe and colleagues (2022). Specifically, Consistency measured the repetition variability in voice onset time (VOT) across the first nine accurate syllables, while Coordination measured gap duration during each syllable transition (Rowe et al., 2022). Further, accuracy on diadochokinetic tasks was measured as percent consonants correct across each participant's first attempt. Intra-rater reliability was excellent across all measures (VOT: ICC(2,1) .967 [.956, .975], < .001; Gap duration: ICC(2,1) .993 [.990, .995], < .001; Accuracy: ICC(2,1) .998 [.996, .999], < .001).

The final motor speech measure was pairwise variability index (PVI), a measure of lexical stress which was calculated based on the median value across participants' productions of seven multisyllabic iambic words (e.g., banana, potato; complete word list in Table S1). PVI, a measure of the relative vowel duration of the weak and strong syllables, was calculated according to previously published methods (Ballard et al., 2016; Duffy et al., 2017; Vergis et al., 2014) based on acoustic measurements of the duration of the first and second vowel in each word. Acoustic analyses were completed by the same trained research assistant, who re-measured 20% of participants with excellent intra-rater reliability on vowel duration measurements: (ICC(2,1) .9990 [.9985, .9994], < .001, absolute agreement on single measures).

Table S1. Pairwise Variability Index Word List. High-quality recordings of productions of the following words were obtained. Words were produced in a multisyllabic word repetition task (Instructions: "Repeat what I say."). *Item was excluded from PVI calculations due to missing data.

Potato

Tomato*

Pajamas

Banana

Catastrophe

Spaghetti

Toboggan

Mechanic

Working memory measure.

The *Digit Span Forward* subtest from the National Alzheimer's Coordinating Center Uniform Data Set neuropsychological test battery (Besser et al., 2018; Weintraub et al., 2009) was also administered during the experimental sessions. Prior work has shown this measure to be highly selective for phonological working memory with less involvement of general cognitive or language regions than other common clinical repetition measures (Miller et al., 2021).

Comprehensive language evaluation.

Patients completed comprehensive speech-language testing with speech-language pathologists in our group within six months of their participation in this speech learning experiment (mean: 52.9 days, SD: 34.2 days; range: 8-157 days). Available language assessment data included the Repetition and Comprehension of Written Sentences subtests from the Western Aphasia Battery-Revised (WAB-R; Kertesz, 2007); the Sentence Comprehension Test from the Northwestern Assessment of Verbs and Sentences (NAVS; Thompson, 2011); the shortened 30-item (odd items) version of the Boston Naming Test (Kaplan et al., 1983) from the National Alzheimer's Coordinating Center Uniform Data Set Version 3 battery; the Word-Picture Matching subtest (first 32 items only) from the Cambridge Semantic Battery (Adlam et al., 2010); the Spelling subtest from the Boston Diagnostic Aphasia Examination (Goodglass et al., 2001); and letter and category fluency tasks (for letter fluency, maximum performance across F, A, and S; and for categories, maximum performance across animals, fruit, and vegetables) from the Controlled Oral Word Association Test (Spreen & Strauss, 1991). Not all clinical measures were administered for each participant, but at least 80% of participants completed each test. Some missing data are due to differences in study protocols, since evaluations were conducted as part of several different longitudinal studies. Some assessments were also not administered if not appropriate for patients' severity levels (i.e., could not complete practice item or did not appear to understand task instructions).

Clinician ratings of severity across a number of language domains were completed by speech-language pathologists in our group according to the Progressive Aphasia Severity Scale (PASS; Sapolsky et al., 2014). These clinician ratings were completed within nine months of the behavioral speech learning

session (mean: 72.1 days, SD: 67.4 days; range: 8-273 days). PASS ratings were not available for one participant. Specifically, PASS ratings in the domains of fluency, articulation, and repetition were included in statistical analyses, as well as the Sum of Boxes score across all language domains.

Supplementary Materials 2: Stimulus Sets

Table S2.

**Stimulus Set**	**Training Items**	**Novel Test Items**		
	zdat	vzat	pkat	kfat
**A**	shfig	fpig	kshig	zbig
	pshud	kpud	gvud	shkud
	vzat	zdat	pkat	kfat
**B**	fpig	shfig	kshig	zbig
	kpud	pshud	gvud	shkud
	pkat	zdat	vzat	kfat
**C**	kshig	shfig	fpig	zbig
	gvud	pshud	kpud	shkud
	kfat	zdat	vzat	pkat
**D**	zbig	shfig	fpig	kshig
	shkud	pshud	kpud	gvud

Supplementary Materials 3: Imaging Cohort Characterization: Demographics, Atrophy Patterns, and Learning Data

Demographics. The 19 participants included in the imaging analyses were slightly younger than the full cohort, with a mean age of 67.5 years (STD = 9.3 years) and a similar balance of males to females (11 males). Participants in the imaging cohort had a similar disease duration as the full cohort (mean = 4.5 years post symptom onset; STD = 2.2). Our imaging cohort included participants from all four diagnostic subgroupings included in the main behavioral analyses (logopenic variant PPA: n = 6, semantic variant PPA: n = 6, nonfluent variant PPA: n = 4, PPA-Other: n = 3). Within the nfvPPA group, two were diagnosed with primary progressive apraxia of speech, one with agrammatism without motor speech impairment, and one with both agrammatism and concomitant AOS. The svPPA, and PPA-Other groups were unchanged from the full behavioral cohort (i.e., imaging data was available for all participants). Mean participant demographics by diagnosis are summarized in Table S1. There were no significant differences between diagnostic subgroups for any of the demographic variables.

Table S3. Demographic and speech/language characteristics by diagnostic group, for nonfluent variant primary progressive aphasia (nfvPPA), semantic variant (svPPA), logopenic variant (lvPPA), PPA-Other. PASS-SB= Progressive Aphasia Severity Score, Sum of Boxes; SD = standard deviation

	**Nonfluent (n=4)**	**Semantic (n=6)**	**Logopenic (n=6)**	**PPA-O (n=3)**
**Age**	65.1 +/ 5.4	63.1 +/ 5.4	69.7 +/ 13.1	75.3 +/ 7.2
**Sex**	2M / 2F (50%)	5M / 1F (83%)	3M / 3F (50%)	1M / 2F (33%)
**Handedness**	3R / 1L (75%)	5R / 1L (83%)	5R / 1L (83%)	3R / 0L (100%)
**PASS- SB**	3.1 +/ 2.1	4.7 +/ 4.3	7.1 +/ 2.5*	2.3 +/ 1.0
**Years Education**	16 +/ 1.6	16.8 +/ 2.9	18.3 +/ 1.5	17 +/ 4.6
**Years Post Onset**	2.6 +/ 0.9	6 +/ 2.4	4.3 +/ 1.8	4.3 +/ 2.1

*Incomplete data (missing 1 lvPPA)

Learning. Spearman correlations confirmed that, within the imaging cohort, error rate and duration were not correlated with each other in either experimental phase (Training measure: $p = .51$; Test measure: $p = .77$). Within the neuroimaging cohort, error rate in the training phase was correlated with the learning measure in the test phase ($r_s = .589$, $p = .008$), while for duration this relationship was marginally significant ($r_s = .468$, $p = .060$); across the imaging cohort, participants with greater improvements in accuracy across the training phase also showed higher accuracy on trained items compared to novel during the test phase.

Atrophy Patterns, by Diagnosis. Structural images were obtained for age-matched control participants (n = 25; mean age = 67.4 years, SD = 4.9; 12 female) using the following scan parameters: TR = 2.3s, TE = 2.95 ms, flip angle = 9.00°, number of inter-leaved sagittal slices = 176, matrix dimensions = 256 x 256, FOV = 270 mm, voxel size = 1.10 mm x 1.10 mm x 1.20 mm. To characterize atrophy for our imaging

cohort compared to controls, separate Mann Whitney U tests were conducted for each ROI using a one-tailed statistical threshold of $p < 0.05$ uncorrected.

Figure S1. Atrophy patterns for all imaging patients, combined across diagnoses, is shown in the top left panel. lvPPA = logopenic variant primary progressive aphasia; nfvPPA = nonfluent variant primary progressive aphasia; PPA-O = primary progressive aphasia, other diagnosis; svPPA = semantic variant primary progressive aphasia

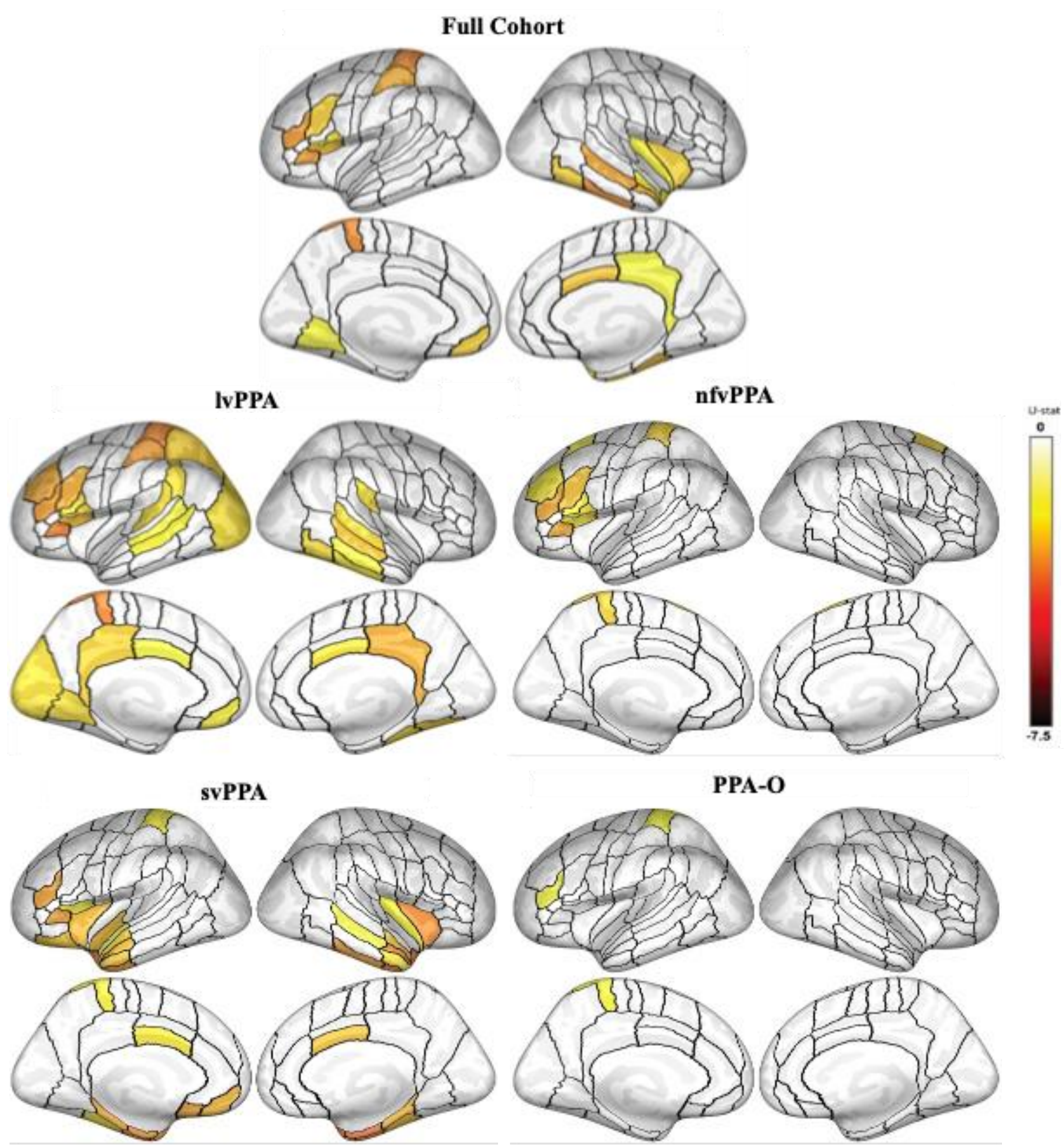

Supplementary Materials 4: Scatter plots for results of *a priori* imaging hypothesis tests.

Scatter plots for correlations between cortical thickness in left posterior inferior frontal sulcus (pIFs) and change in error rate across training phase (top left), error rate improvements in test phase (trained-novel comparison; top right), change in duration across training phase (bottom left), and duration improvements in test phase (trained-novel comparison; bottom right). Significant correlations (uncorrected, $p < .05$) are denoted with an asterisk.

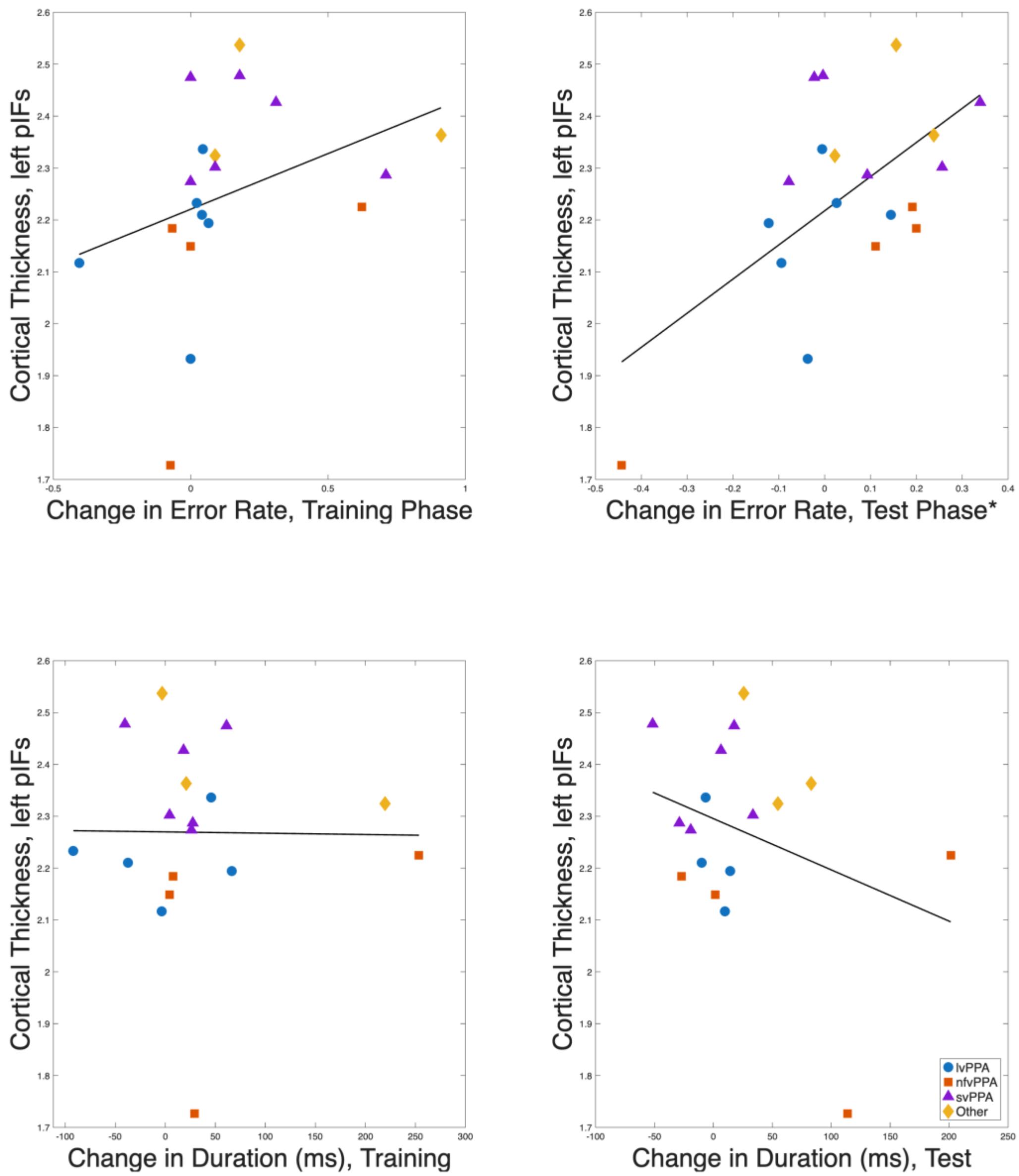

Scatter plots for correlations between cortical thickness in left ventral premotor cortex (vPMC) and change in error rate across training phase (top left), B) error rate improvements in test phase (trained-novel comparison; top right), change in duration across training phase (bottom left), and D) duration improvements in test phase (trained-novel comparison; bottom right). Significant correlations (uncorrected, $p < .05$) are denoted with an asterisk.

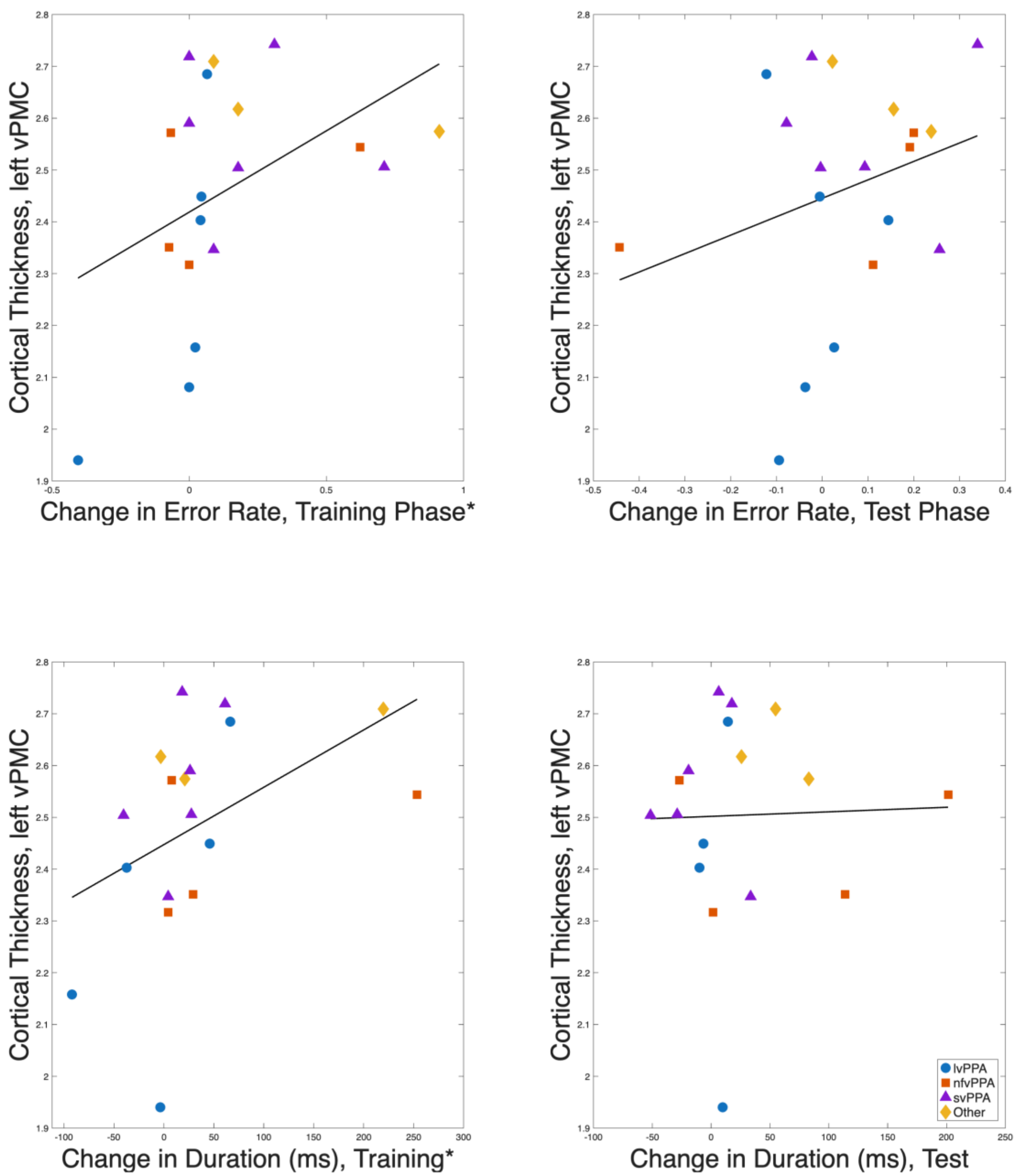

Supplement: Supplementary file 1 [file nol-07-281-s001.pdf]
